# The response of broilers to xylanase and β-glucanase combination in maize-based diets containing wheat distillers’ dried grain with solubles

**DOI:** 10.1016/j.aninu.2025.12.006

**Published:** 2026-03-07

**Authors:** Eunjoo Kim, Mingan Choct, Anna Fickler, Leon Hall, Tamsyn M. Crowley, Nishchal K. Sharma

**Affiliations:** a School of Environmental and Rural Science, University of New England, Armidale 2350, NSW, Australia; b BASF SE, Ludwigshafen 67056, Germany; c Poultry Hub Australia, University of New England, Armidale 2350, NSW, Australia

**Keywords:** Broiler, β-Glucanase, Growth performance, Non-starch polysaccharides, Wheat distillers' dried grain with solubles, Xylanase

## Abstract

Broiler diets containing wheat distillers’ dried grain with solubles (wDDGS) have higher levels of non-starch polysaccharides (NSP) compared to conventional maize-soybean meal-based diets, which can increase intestinal viscosity and impair nutrient utilisation. This study investigated the effects of supplemental xylanase and β-glucanase (XG) preparation on growth performance, ileal flow of soluble NSP constituent sugars, and the gastrointestinal environment in broilers fed maize-based diets with varying wDDGS levels. A total of 768 mixed-sex Cobb 500 broilers (on hatch day and 40 ± 1 g) were assigned to 6 treatments in a 3 × 2 factorial arrangement, with wDDGS levels (none), 6%–12% (medium), 12%–20% (high) with or without XG supplementation across eight replicate pens of 16 birds each, totaling 128 birds per treatment. From d 0 to 10, XG and wDDGS interacted for weight gain (WG; *P* = 0.019), with XG improving WG only in broilers fed high wDDGS diets but not in others. High wDDGS worsened feed conversion ratio (FCR; *P* = 0.014), while XG tended to improve it (*P* = 0.079). From d 21 to 35, XG increased WG (*P* = 0.001) and improved FCR (*P* = 0.006) regardless of wDDGS level. From d 0 to 35, XG increased WG (*P* < 0.001) and tended to improve FCR regardless of wDDGS level (*P* = 0.097). Medium and high wDDGS diets raised ileal viscosity at d 21 (*P* = 0.020) and high wDDGS at d 35 (*P* = 0.002). XG lowered ileal viscosity at d 21 (*P* = 0.006) and d 35 (*P* = 0.001). Medium and high wDDGS diets resulted in higher ileal flow of soluble NSP constituent sugars, including soluble arabinose, mannose, and glucose compared with the control (*P* < 0.001). XG reduced soluble arabinose (*P* = 0.012) and total soluble NSP constituent sugars (*P* = 0.007). Dietary XG and wDDGS interacted for ileal soluble xylose concentration within the NSP fraction (*P* = 0.018), where XG reduced soluble xylose in broilers fed medium and high wDDGS diets but not the control (no wDDGS). High wDDGS diets increased the ileal flow of insoluble NSP constituent sugars vs. the control diet with no wDDGS (*P* < 0.001). XG supplementation did not affect ileal insoluble NSP constituent sugars (*P* > 0.05), but tended to elevate xylose in the free oligosaccharide fraction in the ileum (*P* = 0.053). XG increased caecal propionic acid (*P* = 0.004) and tended to increase caecal short-chain fatty acids (*P* = 0.060). In conclusion, XG supplementation enhanced broiler performance in maize-based diets with wDDGS by reducing soluble NSP and small intestinal viscosity while promoting hindgut fermentation, though optimised dosing is needed for high wDDGS diets to support sustainable broiler nutrition.

## 1 Introduction

Global poultry meat consumption is projected to rise 16% by 2033, accounting for 43% of the total protein consumed from all meat sources (OECD/FAO, 2024). This growth is driven by several factors, with price being a major one, as poultry remains the most affordable meat. However, a key challenge moving forward will be maintaining this price advantage, which largely depends on production costs, particularly feed expenses, which comprise approximately 70% of total production costs. One way to reduce feed costs is to replace some grains and protein meals with alternative ingredients that are not suited for human consumption. Increasing the capacity of poultry to convert alternative ingredients into edible protein will be crucial for sustainably meeting the rising global demand for poultry meat.

Wheat distillers' dried grain with solubles (wDDGS) is a primary byproduct of bioethanol production from wheat and is increasingly used as an alternative feed ingredient for poultry. During the fermentation process of wheat, most of the starch is converted to ethanol, leading to a 2 to 3 fold increase in residual nutrients, such as crude protein (CP), fat, minerals, and vitamins (Nyachoti et al., 2005; Thacker and Widyaratne, 2007), making wDDGS an attractive alternative feed ingredient for poultry. However, the non-starch polysaccharide (NSP) levels in wDDGS are also increased during this process, which limits its use in poultry feed. The total NSP concentration in wDDGS ranges from 24% to 30% dry matter (DM), which includes 3%–7% cellulose, 5%–8% soluble NSP, and the remainder as insoluble NSP. It contains 12%–16% arabinoxylans, of which 2%–6% is soluble, and the rest is insoluble (Pedersen et al., 2014; Widyaratne and Zijlstra, 2007). Including high levels of wDDGS in broiler diets may increase the dietary level of soluble NSP, thus elevating the digesta viscosity to a level that negatively impacts nutrient absorption and growth performance in poultry (Bedford, 1997; Choct and Annison, 1992). In maize-soy-based diets, wDDGS increases soluble NSP, thus elevating intestinal viscosity. In addition, wDDGS may also increase insoluble NSP, which may decrease feed nutritional value by entrapping nutrients in the cell wall matrix, hindering digestion (Choct, 2015; Hetland et al., 2004). Although a small proportion of NSP is fermented by gut microbiota in the distal ileum and caeca, birds lack endogenous enzymes for the effective breakdown of NSP in feed. Thus, exogenous carbohydrases such as xylanase and β-glucanase (XG) may be useful in broiler diets containing wDDGS.

The nutrient and energy availability of wDDGS for poultry vary widely due to differences in parent wheat variety and processing methods (Cozannet et al., 2010; Pedersen et al., 2014; Whiting et al., 2017). This variation coupled with the high levels of NSP in wDDGS limits its use at higher inclusion levels in poultry diets. While low wDDGS inclusion (6% in starter and 12%–15% in grower and finisher diets) is generally considered safe, higher levels compromise growth performance (Lumpkins et al., 2004; Thacker and Widyaratne, 2007). Several studies have demonstrated the adverse effects of high wDDGS inclusion in broiler diets and the advantages of NSP-degrading enzymes. Campasino et al. (2015) observed decreased body weight and feed efficiency in broilers fed starter diets containing 15% wDDGS, with NSP-degrading enzymes improving nutrient digestibility and growth. An in vitro study (Pedersen et al., 2014) reported that xylanase treatments solubilised 80.8%–87.5% of wDDGS pentosans and released 23.5%–27.0% of the total protein, demonstrating the high potential of xylanase to degrade fibre and improve nutrient availability. In our prior work (Kim et al., 2025a), XG in a wheat-based broiler diet resulted in improved weight gain (WG) and feed conversion ratio (FCR) at 35 d of age, primarily through reduced ileal digesta viscosity, increased retention of nutrients, and increased degradation of soluble NSP. These effects of XG are also expected in diets containing high levels of wDDGS, as they contain similar substrates (i.e., arabinoxylans). It was hypothesized that XG supplementation in a maize-based diet with elevated levels of wDDGS would mitigate performance losses by reducing soluble NSP present in wDDGS and intestinal viscosity. This study evaluated XG's effects on growth performance, ileal flow of NSP constituent sugars and the gastrointestinal environment in broilers fed a maize-based diet with varying wDDGS levels.

## 2 Materials and methods

### 2.1 Animal ethics statement

This experiment was conducted with the approval of the Animal Ethics Committee of the University of New England (No. ARA23–054). All broiler management procedures, including health care, husbandry, and animal use, complied with the requirements of the Australian Code for the Care and Use of Animals for Scientific Purposes (NHMRC, 2013).

### 2.2 Housing and bird management

A total of 768 mixed-sex Cobb 500 broiler chicks were obtained from the Baiada hatchery in Tamworth, New South Wales, Australia. On hatch day (d 0), the birds (40 ± 1 g) were randomly assigned to 48 equally sized floor pens (16 birds/pen) in the environment-controlled poultry shed at UNE. Each pen was equipped with a feeder and two nipple drinkers, and birds had ad libitum access to feed and water. Hardwood shavings were spread to a depth of approximately 7 cm in each pen. The room temperature was initially set at 32 to 33 °C and gradually reduced to 22 °C by d 21, where it remained until d 35. The lighting regimen provided 23 h of light at approximately 40 lux on d 1, with darkness increasing by 1 h per d until reaching 6 h of darkness, after which 18 h of light per d at 10 lux was maintained for the remainder of the study.

### 2.3 Diets and experimental design

The diets were thoroughly mixed and cold pelleted at 65 °C, with the starter diets crumbled. Birds received a starter crumble for the first 10 d post–hatch, followed by 3-mm pelleted grower and finisher diets from d 10 to 21 and 21 to 35, respectively. The diets contained maize and soybean meal as major ingredients with different levels of wDDGS as follows: starter–0 (none), 6% (medium), 12% (high); grower–0 (none), 12% (medium), 18% (high), finisher–0 (none), 12% (medium), 20% (high). The ingredient and nutrient composition of the diets are presented in Table 1. All dietary treatments contained phytase (Natuphos E 10000 G, BASF SE Animal Nutrition, Ludwigshafen, Germany) at 1000 phytase units (FTU/kg), with matrix values assigned for energy, amino acids, and minerals. The diets were formulated to meet Cobb 500 nutrient specifications (Cobb-Vantress, 2022). Vitamin and mineral premixes were included according to the manufacturer's recommendations (UNE VM and UNE TM, Rabar Pty Ltd., Beaudesert, Queensland, Australia). All birds were weighed upon arrival and assigned to one of the six treatments in a 3 × 2 factorial arrangement, with the factors of three wDDGS inclusion levels (none, medium, and high) and XG supplementation (with or without). Each treatment consisted of eight replicate pens with 16 birds each, totaling 128 birds per treatment. XG (Natugrain TS, 560 endo-xylanase unit [TXU]/kg and 250 thermostable endo-glucanase unit [TGU]/kg of xylanase and β-glucanase, respectively) was sourced from BASF Australia Ltd. (Queensland, Australia). One TXU is defined as the amount of enzyme that releases 5 μmol of reducing sugars, measured as xylose equivalents per minute from a buffer solution containing 1 g arabinoxylan per 100 mL at pH 3.5 and 40 °C. One TGU is defined as the amount of enzyme that releases 1 μmol of reducing sugars, measured as glucose equivalents per minute from a buffer solution containing 0.714 g β-glucan per 100 mL at pH 3.5 and 40 °C. The enzyme was added at the expense of sand.

**Table 1:** Ingredient and nutrient composition of the experimental diets (%, as-is basis).

Items	Starter (d 0-10)			Grower (d 10-21)			Finisher (d 21-35)		
	Wheat DDGS levels			Wheat DDGS levels			Wheat DDGS levels		
	None	Medium	High	None	Medium	High	None	Medium	High
**Ingredients**									
Maize	61.57	56.88	53.63	67.35	60.52	55.87	70.32	63.22	57.0
Soybean meal	34.50	33.00	30.10	28.80	23.20	21.60	25.90	20.54	18.41
Wheat DDGS	0.00	6.00	12.0	0.00	12.00	18.00	0.00	12.00	20.00
Canola oil	0.50	0.85	0.97	0.54	0.84	1.17	0.81	1.15	1.58
Limestone	0.85	0.92	1.00	0.86	1.02	1.09	0.83	0.98	1.09
Dicalcium phosphate	1.44	1.27	1.12	0.74	0.44	0.28	0.55	0.25	0.03
Salt	0.35	0.26	0.22	0.33	0.23	0.18	0.33	0.23	0.17
Sand	0.01	0.01	0.01	0.01	0.01	0.01	0.01	0.01	0.01
Titanium dioxide	0.00	0.00	0.00	0.50	0.50	0.50	0.50	0.50	0.50
Vitamin premix^1^	0.08	0.08	0.08	0.08	0.08	0.08	0.08	0.08	0.08
Mineral premix^2^	0.10	0.10	0.10	0.10	0.10	0.10	0.10	0.10	0.10
Choline chloride (60%)	0.00	0.01	0.03	0.00	0.04	0.06	0.01	0.06	0.08
L-Lys HCl	0.19	0.21	0.28	0.25	0.38	0.41	0.22	0.34	0.38
DL-Met	0.30	0.30	0.30	0.30	0.31	0.30	0.26	0.27	0.27
L-Thr	0.10	0.10	0.12	0.11	0.14	0.14	0.07	0.11	0.11
L-Val	0.00	0.00	0.02	0.02	0.06	0.06	0.00	0.04	0.04
L-Arg HCl	0.00	0.00	0.01	0.00	0.12	0.14	0.00	0.11	0.14
Phytase^3^	0.01	0.01	0.01	0.01	0.01	0.01	0.01	0.01	0.01
Total	100.00	100.00	100.00	100.00	100.00	100.00	100.00	100.00	100.00
**Calculated nutrients**^4^									
DM	88.10	88.10	87.90	88.00	87.80	87.70	87.90	87.70	87.60
AMEn, MJ/kg	12.27	12.27	12.27	12.55	12.55	12.55	12.76	12.76	12.76
Dig Arg	1.280	1.267	1.224	1.125	1.125	1.125	1.045	1.044	1.044
Dig Lys	1.134	1.134	1.134	1.044	1.044	1.044	0.954	0.954	0.954
Dig Met	0.577	0.571	0.571	0.546	0.545	0.540	0.503	0.502	0.495
Dig Met + Cys	0.846	0.846	0.846	0.792	0.792	0.792	0.738	0.738	0.738
Dig Trp	0.233	0.233	0.225	0.202	0.188	0.187	0.187	0.173	0.172
Dig Ile	0.828	0.825	0.798	0.730	0.681	0.675	0.682	0.635	0.628
Dig Thr	0.774	0.774	0.774	0.702	0.702	0.702	0.630	0.630	0.630
Dig Val	0.865	0.864	0.864	0.792	0.792	0.792	0.729	0.729	0.729
Ca	0.96	0.96	0.96	0.80	0.80	0.80	0.74	0.74	0.74
Available P	0.54	0.54	0.54	0.40	0.40	0.40	0.36	0.36	0.36
Sodium	0.17	0.16	0.16	0.16	0.16	0.16	0.16	0.16	0.16
**Analyzed nutrients**									
DM	87.70	89.40	89.60	89.10	89.50	89.40	89.40	89.60	89.80
CP	21.10	20.60	20.90	18.90	18.90	20.00	18.00	18.60	18.90

DDGS = distillers' dried grain with solubles; DM = dry matter; AMEn = apparent metabolizable energy corrected for nitrogen retention; CP = crude protein.

1 Vitamin premix per kg diet (UNE VM, Rabar Pty Ltd., Beaudesert, Queensland, Australia): vitamin A, 12 MIU; vitamin D, 5 MIU; vitamin E, 75 mg; vitamin K, 3 mg; nicotinic acid, 55 mg; pantothenic acid, 13 mg; folic acid, 2 mg; riboflavin, 8 mg; cyanocobalamin, 0.016 mg; biotin, 0.25 mg; pyridoxine, 5 mg; thiamine, 3 mg; antioxidant, 50 mg.

2 Mineral premix per kg diet (UNE TM, Rabar Pty Ltd., Beaudesert, Queensland, Australia): Cu (copper sulfate), 16 mg; Mn (manganese sulfate), 60 mg; Mn (manganous oxide), 60 mg; I (potassium iodide), 0.125 mg; Se, 0.3 mg; Fe (iron sulfate), 40 mg; Zn (zinc oxide), 50 mg; Zn (zinc sulfate), 50 mg.

3 Natuphos E 10000 G (BASF SE, Ludwigshafen, Germany).

4 The major ingredients maize, soybean meal and wheat DDGS were analysed for crude protein, amino acids and apparent metabolizable energy (AME) by near-infrared spectroscopy (NIRS) (Evonik Industries AG, Essen, Germany) before diet formulation to get the calculated values. The University of New England (Amidale, NSW, Australia) has a robust ingredient database in the feed formulation software for all ingredients.

### 2.4 Growth performance

Birds and feed were weighed upon arrival and on d 10, 21, and 35 on a pen basis. Mortality was recorded daily. The feed intake (FI), WG, and mortality-adjusted FCR were calculated for each growth stage.

### 2.5 Intestinal viscosity, gastrointestinal pH, and litter moisture

On d 21 and 35, four birds per pen, close to the mean body weight, were selected and euthanized by electrical stunning (MEFE CAT 44N, Mitchell Engineering Food Equipment, Clontarf, Queensland, Australia) followed by cervical dislocation. The carcass was dissected to measure gut pH and collect gut contents to assess viscosity.

The pH of the gizzard, ileal, and caecal contents were measured in situ using a pH probe (Hanna FC200, Hanna Instruments, Woonsocket, RI, USA) connected to a pH meter (OHAUS ST-300G, Ohaus, Parsippany, NJ, USA). The probe was inserted directly into the digesta without touching the gut wall. Each measurement was taken twice, and the average pH value was recorded. The probe was rinsed with ultra-pure water (ICW 3000 water purifier for ion chromatography, Millipore Corp., Burlington, MA, USA) between samples. The jejunal and ileal contents were pooled per pen. Briefly, 2 g of thoroughly mixed fresh digesta samples were transferred into 2-mL Eppendorf tubes and centrifuged at 10,000 × *g* for 10 min at room temperature. Viscosity was measured using 0.5 mL of the resulting supernatant with a Brookfield DV3T Rheometer (Brookfield Ametek, Instrumentation & Specialty Controls Division, Middleboro, MA, USA), with CPA-40Z Spindle, at 25 °C. Viscosity measurements were expressed in centipoise (cPs) unit (1 cPs = 1/100 dyne·s per cm^2^ = 1 mPa·s) before statistical analysis. The remaining digesta were stored at −20 °C until further processing. At d 35, litter samples were collected from five different points of individual pens in a plastic zip bag to measure the litter moisture.

### 2.6 Caecal short-chain fatty acids (SCFA) and lactic acid

Short-chain fatty acid and lactic acid concentrations were measured in the caecal digesta samples using the previously described method (Jensen et al., 1995) with some modifications. In brief, 1 mL internal standard (0.01 mol/L ethylbutyric acid) was added to approximately 2 g fresh digesta sample, mixed, and centrifuged at 3900 × *g* at 5 °C for 20 min. Approximately 1 mL the resulting supernatant, 0.5 mL concentrated HCl (36%), and 2.5 mL ether were combined. The mixture was then centrifuged at 2000 × *g* at 5 °C for 15 min and 400 μL the resulting supernatant was combined with 40 mL N-tert-butyldimethylsilyl-N-methyltrifuoroacetamide (MTBSTFA) in a gas chromatograph (GC) vial. The samples were then heated at 80 °C for 20 min, left at room temperature for 48 h, and were analyzed on a Varian CP3400 CX GC (Varian Analytical Instruments, Palo Alto, CA, USA). The SCFA concentration was expressed as μmol/g digesta.

### 2.7 Measurements and chemical analyses

Frozen ileal digesta samples were dried using a freeze-dryer (Alpha 1-4 LDplus, Martin Christ, Osterode am Harz, Lower Saxony, Germany) and ground to pass through a 0.5-mm screen. Ground feed and ileal contents were analyzed in duplicate for titanium dioxide (TiO_2_) following the method described by Short et al. (1996) by UV-spectroscopy at 410 nm (Cary 50 Bio UV–Visible spectrophotometer equipped with a Cary 50 MPR microplate reader (Varian Inc., Palo Alto, CA, USA). Ground feed was analyzed in duplicate for nitrogen using the Dumas method with a LECO FP-2000 automatic N analyzer (Leco Corp., St. Joseph, MI, USA). The nitrogen value was multiplied by 6.25 to get the crude protein value. The DM content of the ground feed, freeze-dried digesta, and litter samples was determined using oven-drying (Thermoline, Thermo Electron Corp., Wetherill Park, Australia) following the method 934.01 (AOAC, 1990). The composition of soluble and insoluble NSP constituent sugars and free oligosaccharides in feed and ileal digesta samples (d 21) was analyzed in duplicate using an Agilent 8890GC equipped with an Agilent 7693A autosampler (Agilent Technologies Inc., Palo Alto, CA, USA). The analysis followed the procedure of Englyst et al. (1994) with modifications as described by Theander et al. (1995) and Morgan et al. (2019).

### 2.8 Calculations

Ileal NSP and oligosaccharide concentrations (g/kg marker DM) were corrected using the TiO_2_ marker concentration in the digesta and diet as follows:

Ileal NSP concentration = NSP constituent sugar _Ileal digesta_ × (TiO_2__Ileal digesta_/TiO_2__Diet_),

Oligosaccharide concentration = Oligosaccharide constituent sugar _Ileal digesta_ × (TiO_2__Ileal digesta_/TiO_2__Diet_).

### 2.9 Statistical analysis

Data were analyzed using the General Linear Model procedures in IBM SPSS statistical software version 28. The pen was considered the experimental unit for statistical analysis. Normality was assessed using the Kolmogorov–Smirnov test. A two-way analysis of covariace (ANCOVA) was performed to evaluate the main effects of wDDGS, XG and their interaction. The statistical model used was as follows:

*Y*_*ij*_*=μ* + *W*_*i*_*+X*_*j*_*+R*_*ij*_ + *ε*_*ij*_,

where *Y*_*ij*_ is the observation of dependent variables; *μ* is the overall mean; *W*_*i*_ is the wDDGS effect; *X*_*j*_ is the XG effect; *R*_*ij*_ represents the interactive effect between the two factors; and *ε*_*ij*_ is the residual error for the observation. The sex ratio of each pen was applied as a covariate in the analysis. When significant differences were detected, means were separated using Tukey's HSD test. Statistical significance was set at *P <* 0.05, while trends towards significance were considered at 0.05 < *P <* 0.10.

## 3 Results

### 3.1 Diet composition

Experimental diets maintained similar energy and nutrient levels across wDDGS inclusion (none, medium: 6%–12%, and high: 12%–20%), but NSP constituent sugars increased with wDDGS (Table 1, Table 2). Medium wDDGS diets contained 1.7 to 2.3 times more soluble NSP and 1.05 to 1.20 times more insoluble NSP, resulting in 8%–23% more total NSP than the control diet with no wDDGS. The high wDDGS diets contained 2.1 to 3.5 times more soluble NSP and 1.1 to 1.3 times more insoluble NSP, resulting in 16%–43% more total NSP than the control diet with no wDDGS. Soluble arabinose and xylose were notably elevated in wDDGS diets, contributing to increased viscosity potential.

**Table 2:** Analysed non-starch polysaccharide constituent sugars (g/kg, DM basis) of the experimental diets.

Constituent sugars	Starter (d 0-10)			Grower (d 10-21)			Finisher (d 21-35)		
	Wheat DDGS levels			Wheat DDGS levels			Wheat DDGS levels		
	None	Medium	High	None	Medium	High	None	Medium	High
Total NSP	75.4	81.6	87.7	75.3	89.4	99.4	74.5	91.7	106.6
**Soluble NSP**	3.6	6.1	7.7	3.7	7.8	9.8	3.6	8.4	12.4
Rhamnose	ND	ND	ND	ND	ND	ND	ND	ND	ND
Fucose	ND	ND	ND	ND	ND	ND	ND	ND	ND
Ribose	ND	ND	ND	ND	0.09	0.10	ND	0.10	0.12
Arabinose	1.1	1.7	2.6	1.0	2.5	3.3	1.0	2.6	3.8
Xylose	0.5	1.4	2.8	0.4	2.5	3.9	0.4	2.9	4.5
Mannose	0.4	0.6	0.5	0.5	0.8	0.7	0.7	0.9	1.3
Galactose	1.2	1.6	1.6	1.3	1.7	1.9	1.1	1.7	2.4
Glucose	0.8	1.5	1.2	0.9	1.3	1.2	0.7	1.3	2.0
**Insoluble NSP**	71.8	75.5	80.0	71.7	81.6	89.5	70.9	83.4	94.2
Rhamnose	ND	ND	ND	ND	ND	ND	ND	ND	ND
Fucose	1.4	1.2	1.2	1.1	0.9	0.9	1.1	0.8	0.8
Ribose	0.68	0.72	0.74	0.68	0.57	0.57	ND	ND	0.58
Arabinose	21.3	23.8	25.4	21.5	24.3	27.2	21.5	25.1	29.5
Xylose	22.3	27.2	30.9	23.3	28.4	35.2	24.1	30.9	37.4
Mannose	4.0	4.1	3.9	4.4	4.5	4.7	3.9	4.4	4.8
Galactose	19.8	18.6	17.6	17.8	14.5	14.7	17.0	14.1	13.8
Glucose	11.1	9.4	10.3	11.9	18.9	17.6	12.3	18.5	19.4
**Oligosaccharide**	49.7	49.7	51.5	45.6	49.5	54.7	44.2	46.9	50.5
Rhamnose	ND	ND	ND	ND	ND	ND	ND	ND	ND
Fucose	ND	ND	ND	ND	ND	ND	ND	ND	ND
Ribose	ND	ND	ND	ND	ND	ND	ND	ND	ND
Arabinose	0.6	1.1	1.6	0.6	1.5	2.0	0.5	1.6	2.2
Xylose	ND	1.0	2.2	ND	2.2	3.5	ND	2.4	3.8
Mannose	7.2	6.4	7.2	5.8	6.7	7.9	6.0	6.3	6.0
Galactose	11.3	11.2	10.5	10.4	9.1	8.9	9.9	8.8	8.6
Glucose	30.5	30.0	30.0	28.7	29.9	32.3	27.8	27.8	29.9

NSP = Non-starch polysaccharides; DDGS = distillers' dried grain with solubles; ND = not detected.

### 3.2 Growth performance

The wDDGS and XG significantly influenced growth performance (Table 3). From d 0 to 10, XG and wDDGS interacted for WG (*P* = 0.019), with XG improving gain in high wDDGS diets (12%) to match control levels but not in others. High wDDGS worsened FCR (*P* = 0.014), while XG tended to improve it (*P* = 0.079). Over d 10 to 21 (*P* = 0.006), d 21 to 35 (*P* < 0.001), and d 0 to 35 (*P* = 0.011), medium and high wDDGS impaired FCR, and high wDDGS reduced WG (*P* < 0.001) compared to the control. XG enhanced WG by 5.0% (*P* = 0.001) and improved FCR by 7 points (*P* = 0.006) from d 21 to 35, and increased WG by 3.5% (*P* < 0.001) with a trend for better FCR (*P* = 0.097) over d 0 to 35, regardless of wDDGS level. FI was unaffected by XG (*P* > 0.05). Mortality remained low (<3%) and was not affected by dietary treatment (data not shown).

**Table 3:** Effects of in-feed carbohydrase on growth performance of broilers.

Items		Starter (d 0-10)			Grower (d 10-21)			Finisher (d 21-35)			Overall (d 0-35)		
		WG, g	FI, g	FCR, g/g	WG, g	FI, g	FCR, g/g	WG, g	FI, g	FCR, g/g	WG, g	FI, g	FCR, g/g
**wDDGS**	**XG**												
None	–	225^a^	256	1.14	718	940	1.31	1399	2082	1.49	2343	3278	1.40
	+	227^a^	251	1.10	719	949	1.32	1488	2133	1.44	2434	3333	1.37
Medium	–	222^a^	251	1.13	675	938	1.39	1377	2259	1.65	2274	3166	1.52
	+	225^a^	257	1.14	700	938	1.34	1465	2296	1.57	2389	3491	1.46
High	–	199^b^	237	1.19	676	924	1.37	1210	1894	1.63	2084	3056	1.51
	+	221^a^	251	1.14	658	902	1.37	1233	1992	1.57	2111	3146	1.45
SEM		2.0	2.0	0.007	5.2	7.1	0.008	17.9	34.8	0.015	20.9	58.0	0.017
**Main effects**													
wDDGS	None	226	253	1.12^b^	718^a^	945	1.32^b^	1443^a^	2107^ab^	1.46^b^	2388^a^	3305	1.38^b^
	Medium	224	254	1.13^b^	687^b^	938	1.36^a^	1421^a^	2278^a^	1.61^a^	2332^a^	3328	1.49^a^
	High	210	244	1.17^a^	667^b^	913	1.37^a^	1221^b^	1943^b^	1.60^a^	2098^b^	3100	1.48^a^
XG	–	215	248	1.15	689	934	1.36	1328	2111	1.59	2234	3199	1.48
	+	224	253	1.13	692	930	1.34	1395	2108	1.52	2311	3290	1.43
*P-*value	wDDGS	<0.001	0.064	0.014	<0.001	0.170	0.006	<0.001	<0.001	<0.001	<0.001	0.213	0.011
	XG	0.007	0.162	0.079	0.740	0.759	0.393	0.001	0.954	0.006	<0.001	0.429	0.097
	wDDGS × XG	0.019	0.105	0.133	0.130	0.653	0.195	0.280	0.526	0.871	0.095	0.310	0.874

XG = a xylanase and β-glucanase preparation; wDDGS = wheat distillers' dried grain with solubles; WG = weight gain; FI = feed intake; FCR = mortality corrected feed conversion ratio; + = with; - = without; SEM = standard error of the mean.

Within a column, means without a common superscript letter differ at *P* < 0.05, *n* = 8.

### 3.3 Intestinal viscosity and litter moisture

As presented in Table 4, medium and high wDDGS diets increased ileal viscosity on d 21 (*P* = 0.020) and jejunal viscosity on d 35 (*P* = 0.004) compared to the control. Additionally, high wDDGS increased jejunal viscosity on d 21 (*P* = 0.016) and ileal viscosity on d 35 (*P* = 0.002) compared to the control. XG reduced ileal viscosity on d 21 (*P* = 0.006) and d 35 (*P* = 0.001), mitigating wDDGS effects. High wDDGS elevated litter moisture (*P* = 0.001), which XG decreased (*P* = 0.047).

**Table 4:** Effects of in-feed carbohydrase on intestinal viscosity (cPs) and litter moisture (%).

Items		d 21		d 35		
		Jejunum	Ileum	Jejunum	Ileum	Litter moisture
**wDDGS**	**XG**					
None	–	1.91	2.96	2.68	3.13	41.6
	+	1.88	2.67	2.16	2.86	40.5
Medium	–	1.94	3.21	3.17	3.24	47.0
	+	1.97	2.94	3.02	3.08	42.3
High	–	2.03	3.10	2.98	3.92	48.1
	+	2.02	2.97	3.14	3.14	46.2
SEM		0.180	0.045	0.967	0.073	0.84
**Main effects**						
wDDGS	None	1.90^b^	2.81^b^	2.42^b^	2.99^b^	41.1^b^
	Medium	1.95^ab^	3.08^a^	3.09^a^	3.16^ab^	44.7^ab^
	High	2.02^a^	3.03^a^	3.06^a^	3.53^a^	47.2^a^
XG	–	1.96	3.09	2.94	3.43	45.6
	+	1.96	2.86	2.78	3.03	43.0
*P-*value	wDDGS	0.016	0.020	0.004	0.002	0.001
	XG	0.990	0.006	0.346	0.001	0.047
	wDDGS × XG	0.806	0.673	0.284	0.083	0.489

XG = a xylanase and β-glucanase preparation; wDDGS = wheat distillers' dried grain with solubles; + = with; - = without; SEM = standard error of the mean.

Within a column, means without a common superscript letter differ at *P* < 0.05, *n* = 8.

### 3.4 Gastrointestinal environment

The wDDGS and XG had limited effects on gastrointestinal pH (Table 5). High wDDGS reduced caecal pH on d 21 (*P* = 0.010), and an interaction between XG and wDDGS further lowered caecal pH in high wDDGS diets on d 35 (*P* = 0.005). Gizzard and ileal pH were unaffected (*P* > 0.05).

**Table 5:** Effects of in-feed carbohydrase on gastrointestinal pH.

Items		d 21			d 35		
		Gizzard	Ileum	Caeca	Gizzard	Ileum	Caeca
**wDDGS**	**XG**						
None	–	2.54	6.33	6.66	3.05	7.01	6.79^a^
	+	2.57	6.17	6.73	2.99	6.96	6.67^ab^
Medium	–	2.40	6.30	6.43	2.64	7.06	6.42^b^
	+	2.61	6.19	6.55	3.00	6.78	6.57^ab^
High	–	2.40	6.37	6.51	2.85	6.85	6.77^a^
	+	2.34	6.35	6.39	2.83	6.98	6.41^b^
SEM		0.039	0.038	0.036	0.052	0.039	0.036
**Main effects**							
wDDGS	None	2.55	6.25	6.69^a^	3.02	6.98	6.73
	Medium	2.51	6.25	6.49^ab^	2.82	6.92	6.49
	High	2.37	6.36	6.45^b^	2.84	6.91	6.59
XG	–	2.45	6.33	6.53	2.85	6.97	6.66
	+	2.51	6.24	6.55	2.94	6.91	6.55
*P-*value	wDDGS	0.130	0.384	0.010	0.351	0.696	0.010
	XG	0.428	0.223	0.755	0.311	0.379	0.076
	wDDGS × XG	0.334	0.742	0.334	0.396	0.107	0.005

XG = a xylanase and β-glucanase preparation (Natugrain TS); wDDGS = wheat distillers' dried grain with solubles; + = with; - = without; SEM = standard error of the mean.

Within a column, means without a common superscript letter differ at *P* < 0.05, *n* = 8.

### 3.5 Ileal flow of soluble NSP

Medium and high wDDGS increased the ileal flow of soluble NSP constituent sugars, including arabinose, mannose, and glucose (*P* < 0.001), and high wDDGS raised soluble galactose (*P* < 0.001) compared to the control (Table 6). XG reduced soluble arabinose (*P* = 0.012) and total soluble NSP constituent sugars (*P* = 0.007), and interacted with wDDGS (*P* = 0.018) to decrease soluble xylose in medium and high wDDGS diets. High wDDGS increased insoluble NSP constituent sugars (*P* < 0.001), including arabinose, xylose, and glucose (*P* < 0.001), unaffected by XG (*P* > 0.05, Table 7).

**Table 6:** Effects of in-feed carbohydrase on ileal flow (g/kg marker DM) of soluble non-starch polysaccharide constituent sugars at 21 d.

Items		Constituent sugars								
		Rhamnose	Fucose	Ribose	Arabinose	Xylose	Mannose	Galactose	Glucose	Total
**wDDGS**	**XG**									
None	–	0.03	0.34	0.00	1.7	0.8^e^	0.4	2.1	0.4	5.2
	+	0.01	0.34	0.00	1.6	0.6^e^	0.3	1.9	0.3	4.6
Medium	–	0.05	0.32	0.00	3.0	3.0^c^	0.8	2.4	1.0	9.3
	+	0.04	0.29	0.00	2.5	2.0^d^	0.7	2.2	1.0	7.8
High	–	0.04	0.35	0.01	4.2	4.9^a^	1.0	2.9	1.3	13.1
	+	0.05	0.38	0.00	3.9	3.9^b^	1.0	2.8	1.4	11.9
SEM		0.005	0.007	0.001	0.15	0.24	0.05	0.07	0.07	0.50
**Main effects**										
wDDGS	None	0.02	0.34^ab^	0.00	1.6^c^	0.7	0.4^c^	2.0^b^	0.3^c^	4.9^c^
	Medium	0.05	0.30^b^	0.00	2.7^b^	2.5	0.8^b^	2.3^b^	1.0^b^	8.5^b^
	High	0.04	0.36^a^	0.00	4.0^a^	4.4	1.0^a^	2.8^a^	1.3^a^	12.5^a^
XG	–	0.04	0.34	0.00	2.9	2.9	0.8	2.4	0.9	9.2
	+	0.03	0.33	0.00	2.7	2.2	0.7	2.3	0.9	8.1
*P-*value	wDDGS	0.097	0.002	0.347	<0.001	<0.001	<0.001	<0.001	<0.001	<0.001
	XG	0.679	0.924	0.272	0.012	<0.001	0.216	0.295	0.952	0.007
	wDDGS × XG	0.653	0.287	0.300	0.281	0.018	0.870	0.811	0.469	0.643

DM = dry matter; XG = a xylanase and β-glucanase preparation (Natugrain TS); wDDGS = wheat distillers' dried grain with solubles; + = with; - = without; SEM = standard error of the mean.

Within a column, means without a common superscript letter differ at *P* < 0.05, *n* = 8.

**Table 7:** Effects of in-feed carbohydrase on ileal flow (g/kg marker DM) of insoluble non-starch polysaccharide constituent sugars at 21 d.

Items		Constituent sugars								
		Rhamnose	Fucose	Ribose	Arabinose	Xylose	Mannose	Galactose	Glucose	Total
**wDDGS**	**XG**									
None	–	0.71	1.4	ND	25.3	28.3	1.8	19.3	3.3	71.0
	+	0.78	1.5	ND	25.5	28.2	1.7	20.5	3.3	72.4
Medium	–	0.62	1.2	ND	25.1	31.3	2.0	16.1	4.4	72.3
	+	0.53	1.1	ND	26.1	30.7	1.8	15.1	4.4	69.8
High	–	0.54	1.3	ND	33.5	42.0	2.3	18.0	5.6	91.4
	+	0.63	1.3	ND	32.0	39.7	2.3	17.3	5.8	87.6
SEM		0.025	0.03	ND	0.56	0.86	0.06	0.41	0.23	1.41
**Main effects**										
wDDGS	None	0.74^a^	1.4^a^	ND	25.4^b^	28.3^c^	1.8^b^	19.9^a^	3.3^b^	71.7^b^
	Medium	0.57^b^	1.1^b^	ND	25.6^b^	31.0^b^	1.9^b^	15.6^b^	4.4^b^	71.0^b^
	High	0.58^b^	1.3^a^	ND	32.8^a^	40.9^a^	2.3^a^	17.6^b^	5.7^a^	89.5^a^
XG	–	0.62	1.3	ND	28.0	33.9	2.0	17.8	4.4	78.2
	+	0.65	1.3	ND	27.9	32.9	1.9	17.7	4.5	76.6
*P-*value	wDDGS	0.005	<0.001	ND	<0.001	<0.001	0.005	<0.001	<0.001	<0.001
	XG	0.568	0.770	ND	0.869	0.147	0.476	0.843	0.792	0.227
	wDDGS × XG	0.250	0.416	ND	0.126	0.392	0.883	0.341	0.967	0.276

DM = dry matter; XG = a xylanase and β-glucanase preparation; wDDGS = wheat distillers' dried grain with solubles; + = with; - = without; ND = not detected; SEM = standard error of the mean.

Within a column, means without a common superscript letter differ at *P* < 0.05, *n* = 8.

### 3.6 Ileal flow of insoluble NSP

High wDDGS diets significantly increased the ileal flow of insoluble NSP constituent sugars, including arabinose, xylose, glucose (*P* < 0.001), and mannose (*P* = 0.005), compared to the control (no wDDGS), reflecting higher dietary insoluble NSP content (Table 7). Medium and high wDDGS diets reduced insoluble rhamnose (*P* = 0.005) and galactose (*P* < 0.001). XG supplementation had no effect on insoluble NSP constituent sugars (*P* > 0.05), consistent with its primary action on soluble NSP (Table 2).

### 3.7 Ileal flow of free oligosaccharides

High wDDGS diets increased the ileal flow of free oligosaccharides constituent sugars, including arabinose, xylose, and total oligosaccharides (*P* < 0.001), compared to medium and no wDDGS diets (Table 8). The XG supplementation tended to increase xylose in the free oligosaccharide fraction (*P* = 0.053).

**Table 8:** Effects of in-feed carbohydrase on ileal flow (g/kg marker DM) of oligosaccharide constituent sugars at 21 d.

Items		Constituent sugars								
		Rhamnose	Fucose	Ribose	Arabinose	Xylose	Mannose	Galactose	Glucose	Total
**wDDGS**	**XG**									
None	–	0.29	ND	0.10	0.5	0.3	1.8	8.9	14.1	26.0
	+	0.31	ND	0.12	0.3	0.2	2.0	9.5	15.0	27.7
Medium	–	0.22	ND	0.14	1.0	1.4	1.8	7.1	11.7	23.4
	+	0.17	ND	0.13	1.2	2.0	1.8	7.6	13.5	26.2
High	–	0.15	ND	0.15	1.8	2.5	2.3	8.5	16.8	32.1
	+	0.20	ND	0.17	1.8	3.0	2.1	8.0	15.9	31.1
SEM		0.014		0.007	0.09	0.17	0.06	0.23	0.53	0.79
**Main effects**										
wDDGS	None	0.30^a^	ND	0.11^b^	0.5^c^	0.3^c^	1.9^ab^	9.2^a^	14.6^ab^	26.9^b^
	Medium	0.19^b^	ND	0.13^ab^	1.1^b^	1.7^b^	1.8^b^	7.4^b^	12.6^b^	24.8^b^
	High	0.17^b^	ND	0.16^a^	1.8^a^	2.8^a^	2.2^a^	8.2^ab^	16.4^a^	31.6^a^
XG	–	0.22	ND	0.13	1.1	1.4	1.9	8.2	14.2	27.2
	+	0.23	ND	0.14	1.2	1.7	2.0	8.4	14.8	28.4
*P-*value	wDDGS	<0.001	ND	0.011	<0.001	<0.001	0.008	0.003	0.013	<0.001
	XG	0.711	ND	0.283	0.573	0.053	0.645	0.660	0.567	0.646
	wDDGS × XG	0.274	ND	0.542	0.684	0.193	0.277	0.471	0.542	0.719

DM = dry matter; XG = a xylanase and β-glucanase preparation; wDDGS = wheat distillers' dried grain with solubles; + = with; - = without; ND, not detected; SEM = standard error of the mean.

Within a column, means without a common superscript letter differ at *P* < 0.05, *n* = 8.

### 3.8 Caecal SCFA

Medium and high wDDGS diets increased caecal acetic acid (*P* = 0.041) but reduced isobutyric (*P* = 0.014), valeric (*P* = 0.005), and isovaleric acids (*P* < 0.001) compared to the control (no wDDGS, Table 9). The XG supplementation significantly increased propionic acid (*P* = 0.004) and tended to elevate total SCFA (*P* = 0.060). No XG × wDDGS interaction was observed (*P* > 0.05) for any SCFA in the caecal content.

**Table 9:** Effects of in-feed carbohydrases on caecal short-chain fatty acid (SCFA) and lactic acid of broilers on d 21 (μmol/g).

Items		Constituent SCFA and lactic acid							
		Acetic	Propionic	Butyric	Isobutyric	Valeric	Isovaleric	Total SCFA	Lactic
**wDDGS**	**XG**								
None	–	58.92	6.47	16.56	0.76	1.01	0.23	83.9	0.60
	+	64.80	7.67	15.03	0.83	0.97	0.23	89.5	1.20
Medium	–	65.43	5.82	14.21	0.66	0.77	0.17	87.0	0.78
	+	69.31	7.29	15.44	0.66	0.87	0.17	93.7	0.79
High	–	68.83	5.92	15.59	0.55	0.85	0.12	91.9	0.95
	+	69.37	7.38	16.51	0.64	0.81	0.14	94.8	0.82
SEM		1.236	0.238	0.338	0.029	0.024	0.011	1.36	0.101
**Main effects**									
wDDGS	None	61.86^b^	7.07	15.79	0.80^a^	0.99^a^	0.23^a^	86.7	0.90
	Medium	67.37^a^	6.56	14.83	0.66^bc^	0.82^b^	0.17^bc^	90.4	0.79
	High	69.10^a^	6.65	16.05	0.59^c^	0.83^b^	0.13^c^	93.3	0.89
XG	–	64.39	6.07	15.45	0.66	0.88	0.17	87.6	0.78
	+	67.83	7.45	15.66	0.71	0.88	0.18	92.7	0.94
*P-*value	wDDGS	0.041	0.610	0.301	0.014	0.005	<0.001	0.133	0.887
	XG	0.152	0.004	0.760	0.337	0.902	0.768	0.060	0.444
	wDDGS × XG	0.648	0.962	0.194	0.828	0.330	0.911	0.840	0.318

XG = a xylanase and β-glucanase preparation; wDDGS = wheat distillers' dried grain with solubles; + = with; - = without; SEM = standard error of the mean.

## 4 Discussion

The inclusion of wDDGS increases the NSP content in maize-based broiler diets, significantly elevating intestinal viscosity and impairing nutrient utilization. This study found that medium (6%–12%) and high (12%–20%) wDDGS diets contained soluble NSP levels approximately double and 2.7 times higher, respectively, compared to the control. These diets increased ileal flow of soluble NSP constituent sugars reflected by higher concentrations of arabinose (from 1.6 to 4.0 g/kg), mannose (from 0.4 to 1.0 g/kg), glucose (from 0.3 to 1.3 g/kg), and xylose (from 0.7 to 4.4 g/kg), indicating elevated levels of viscous polysaccharides like arabinoxylans. This led to increased jejunal and ileal viscosity, resulting in a 10 to 11 points poorer overall FCR and a 12% reduction in WG for high wDDGS diets compared to the control. These findings align with prior research indicating that soluble NSP, particularly arabinoxylans, increases digesta viscosity, restricting endogenous enzyme access to substrates and hindering nutrient absorption (Bedford, 1997; Choct, 2015; Choct and Annison, 1992). Our previous study (Kim et al., 2025b) found that XG supplementation had a negligible impact on reducing intestinal viscosity in broilers fed maize-based diets, attributed to their low soluble NSP content compared to viscous grains like wheat or barley. The current study highlights that wDDGS's soluble arabinoxylan content, approximately three times higher than wheat (Pedersen et al., 2014; Widyaratne and Zijlstra, 2007), exacerbates viscosity in maize-based diets.

The XG supplementation effectively mitigated these antinutritive effects by degrading soluble NSP polymers, significantly reducing ileal viscosity and soluble NSP flow. Specifically, XG decreased ileal concentrations of soluble NSP constituent sugars (from 9.2 to 8.1 g/kg), including arabinose (from 2.9 to 2.7 g/kg), and interacted with wDDGS to reduce xylose in medium (from 3.0 to 2.0 g/kg) and high (from 4.9 to 3.9 g/kg) wDDGS diets, indicating targeted hydrolysis of arabinoxylan-rich NSP. This led to significant performance improvements, with a 3.5% increase in WG and a tendency to improve FCR across wDDGS levels over 0 to 35 d. In the starter phase (d 0-10), XG and wDDGS interacted, restoring WG in high wDDGS diets to control levels, enabling inclusion beyond traditional limits of 6% in starter and 12%–15% in grower-finisher diets (Lumpkins et al., 2004; Thacker and Widyaratne, 2007). These results corroborate Campasino et al. (2015), who reported improved nutrient digestibility and growth in broilers fed 15% wDDGS diets with NSP-degrading enzymes, and Whiting et al. (2017), who noted enhanced nutrient availability with enzyme supplementation. An in vitro study by Pedersen et al. (2014) further demonstrated xylanase's ability to solubilize 80.8%–87.5% of wDDGS pentosans and release 23.5%–27.0% of total protein, underscoring XG's efficacy in degrading viscous NSP and improving nutrient availability. As observed in our previous study (Kim et al., 2025b), during the starter phase, XG supplementation in a maize-soy-based control diet did not affect growth performance, but XG consistently enhanced WG and FCR during the finisher phase. This suggests that early release of prebiotic-like oligosaccharides, such as arabinoxylooligosaccharides (AXOS) from long-chain arabinoxylans, may have facilitated chick adaptation to fibrous diets, enabling more effective degradation of complex substrates later (Bedford, 2025).

The XG's benefits extended to the gastrointestinal environment. The XG supplementation increased caecal propionic acid and tended to elevate total SCFA concentrations, indicating enhanced hindgut fermentation. This is likely driven by the breakdown of soluble polymeric arabinoxylans into fermentable, lower molecular weight oligosaccharides, as evidenced by increased ileal flow of arabinose and xylose fractions. This process can selectively stimulate the hindgut microbiota to promote fibre fermentation and produce SCFA (Courtin et al., 2008; Kim et al., 2025a; Morgan et al., 2017). The lower caecal pH in high wDDGS diets with XG further supports increased SCFA production, suggesting prebiotic effects of AXOS on beneficial microbiota. Our previous study (Kim et al., 2025a) reported similar benefits in wheat-based broiler diets, where XG reduced ileal viscosity, increased nutrient retention, and enhanced soluble NSP degradation, indicating that wDDGS's arabinoxylan-rich NSP provides comparable substrates for XG hydrolysis. Medium and high wDDGS diets also increased caecal acetic acid but reduced isobutyric, valeric, and isovaleric acids, reflecting altered microbial fermentation due to higher NSP availability, though XG's primary impact was on propionic acid, a key gut health indicator.

Dietary XG supplementation also addressed environmental challenges. High wDDGS diets increased litter moisture, consistent with Bedford (2006), who linked elevated soluble NSP to wet litter and poor poultry house conditions. XG significantly reduced litter moisture, likely by decreasing intestinal viscosity and improving water retention, offering practical benefits for flock management and environmental sustainability. Furthermore, medium and high wDDGS diets contained 12% and 23% more insoluble NSP, respectively, increasing the ileal flow of insoluble NSP constituent sugars, including arabinose, xylose, and glucose. This may reduce feed nutritional value by trapping nutrients in the cell wall matrix, creating a physical barrier to digestion (Hetland et al., 2004). The XG had no effect on insoluble NSP constituent sugars, as expected given its specificity for soluble NSP, but its mitigation of soluble NSP effects indirectly improved nutrient utilization.

Despite these benefits, XG's efficacy was reduced at high wDDGS levels (20%), with WG improvements (about 30 g) smaller than in control or medium diets (about 100 g). This highlights that XG, at 560 TXU/kg, could effectively improve body weight when wDDGS was used at a moderate level; however, the negative impacts of high wDDGS inclusion appeared to outweigh the benefits of supplemental XG. It is possible that the recommended dosage of XG was insufficient to fully counteract the antinutritive impacts of wDDGS NSP when included at up to 20%. These findings underscore the need for further research into the interactive effects of xylanase dosage and wDDGS inclusion to optimize XG application based on dietary wDDGS levels, which could lead to more sustainable broiler feed formulations. Future studies should investigate combining XG with other carbohydrases, such as β-mannanases or cellulases, to target a broader range of NSP substrates, or increasing XG doses to enhance degradation in high wDDGS diets, potentially improving performance and nutrient utilization.

Dietary XG supplementation in maize-wDDGS based diets reduced soluble NSP and intestinal viscosity, improved growth performance, promoted hindgut fermentation, and mitigated environmental issues like wet litter. By enabling wDDGS inclusion up to 12% in starter and 20% in finisher diets, XG supports cost-effective and sustainable poultry nutrition. However, its reduced efficacy at high wDDGS levels suggests that dose optimization or enzyme combinations are needed to maximize benefits, warranting further investigation.

## 5 Conclusion

Including wDDGS in maize-based broiler diets reduced body WG and feed efficiency mainly due to increased small intestinal digesta viscosity from elevated soluble NSP content, which hindered nutrient utilisation. This was reflected in the high litter moisture in birds fed a high wDDGS diet. The XG supplementation effectively countered these antinutritive effects of wDDGS by reducing intestinal viscosity, while lowering litter moisture and enhancing caecal SCFA production. As a result, broilers maintained their growth performance comparable to those fed the control diet. However, the benefits of XG were diminished when wDDGS was used at inclusion rates beyond the recommendation. Future research should investigate combining multiple carbohydrases at varying doses to optimize substrate degradation in maize-soy-wDDGS diets, further improving broiler growth performance.

## Credit Author Statement

**Eunjoo Kim:** Writing – review & editing, Methodology, Investigation, Formal analysis, Data curation. **Mingan Choct:** Writing – review & editing, Validation, Supervision, Methodology, Investigation, Funding acquisition, Conceptualization. **Anna Fickler:** Writing – review & editing, Resources, Methodology. **Leon Hall:** Writing – review & editing, Resources, Project administration. **Tamsyn M. Crowley:** Writing – review & editing, Supervision, Resources, Project administration. **Nishchal K. Sharma:** Writing – original draft, Methodology, Formal analysis, Data curation.

## Declaration of competing interest

The authors declare the following financial interests/personal relationships which may be considered as potential competing interests: Anna Fickler and Leon Hall are currently employed by BASF SE (Ludwigshafen, Germany). Mingan Choct is an Editors-in-Chief for Animal Nutrition and was not involved in the editorial review or the decision to publish this article. The authors declare the following financial interests which may be considered as potential competing interests: the research project is funded by BASF SE Animal Nutrition, Germany.

## References

[bib1] AOAC (1990).

[bib2] Bedford M.R. (1997). Enzyme in poultry and swine nutrition.

[bib3] Bedford M.R., Perry G.C. (2006). Avian gut function in health and disease.

[bib4] Bedford M.R. (2025). Priming young birds for gut health and resilience. Proc Aust Poult Sci Symp.

[bib5] Campasino A., Williams M., Latham R., Bailey C.A., Brown B., Lee J.T. (2015). Effects of increasing dried distillers' grains with solubles and non-starch polysaccharide degrading enzyme inclusion on growth performance and energy digestibility in broilers. J Appl Poultry Res.

[bib6] Choct M. (2015). LII Simposio Científico de Avicultura. Málaga.

[bib7] Choct M., Annison G. (1992). Anti-nutritive effect of wheat pentosans in broiler chickens: roles of viscosity and gut microflora. Br Poult Sci.

[bib8] Cobb-Vantress (2022). https://www.cobbgenetics.com/assets/Cobb-Files/2022-Cobb500-Broiler-Performance-Nutrition-Supplement.pdf.

[bib9] Courtin C.M., Broekaert W.F., Swennen K., Lescroart O., Onagbesan O., Buyse J., Decuypere E., Van de Wiele T., Marzorati M., Verstraete W., Huyghebaert G., Delcour J.A. (2008). Dietary inclusion of wheat bran arabinoxylooligosaccharides induces beneficial nutritional effects in chickens. Cereal Chem.

[bib10] Cozannet P., Lessire M., Gady C., Metayer J.P., Primot Y., Skiba F., Noblet J. (2010). Energy value of wheat dried distillers grains with solubles in roosters, broilers, layers, and turkeys. Poult Sci.

[bib11] Englyst H.N., Quigley M.E., Hudson G.J. (1994). Determination of dietary fibre as non-starch polysaccharides with gas–liquid chromatographic, high-performance liquid chromatographic or spectrophotometric measurement of constituent sugars. Analyst.

[bib12] Hetland H., Choct M., Svihus B. (2004). Role of insoluble non-starch polysaccharides in poultry nutrition. Worlds Poult Sci J.

[bib13] Jensen M.T., Cox R.P., Jensen B.B. (1995). Microbial production of skatole in the hind gut of pigs given different diets and its relation to skatole deposition in backfat. Anim Sci.

[bib15] Kim E., Choct M., Fickler A., Guilherme A.M.P., Hall L., Crowley T.M., Sharma N.K. (2025). Supplementation of β-mannanase alone or in combination with xylanase and β-glucanase enhanced growth performance, non-starch polysaccharide degradation, and gastrointestinal environment of broilers offered wheat-based diets. Anim Nutr.

[bib16] Kim E., Choct M., Fickler A., Guilherme A.M.P., Hall L., Crowley T.M., Sharma N.K. (2025). Strategic use of β-mannanase, and xylanase and β-glucanase preparation in maize-based diets to improve broiler performance. Anim Nutr.

[bib17] Lumpkins B.S., Batal A.B., Dale N.M. (2004). Evaluation of distillers dried grains with solubles as a feed ingredient for broilers. Poult Sci.

[bib18] Morgan N.K., Wallace A., Bedford M.R., Choct M. (2017). Efficiency of xylanases from families 10 and 11 in production of xylo-oligosaccharides from wheat arabinoxylans. Carbohydr Polym.

[bib19] Morgan N.K., Keerqin C., Wallace A., Wu S.-B., Choct M. (2019). Effect of arabinoxylo-oligosaccharides and arabinoxylans on net energy and nutrient utilization in broilers. Anim Nutr.

[bib20] NHMRC (2013). The national health and medical research council.

[bib21] Nyachoti C.M., House J.D., Slominski B.A., Seddon I.R. (2005). Energy and nutrient digestibilities in wheat dried distillers' grains with solubles fed to growing pigs. J Sci Food Agric.

[bib22] OECD/FAO (2024).

[bib23] Pedersen M.B., Dalsgaard S., Knudsen K.B., Yu S., Lærke H.N. (2014). Compositional profile and variation of distillers dried grains with solubles from various origins with focus on non-starch polysaccharides. Anim Feed Sci Technol.

[bib24] Short F.J., Gorton P., Wiseman J., Boorman K.N. (1996). Determination of titanium dioxide added as an inert marker in chicken digestibility studies. Anim Feed Sci Technol.

[bib25] Thacker P.A., Widyaratne G.P. (2007). Nutritional value of diets containing graded levels of wheat distillers grains with solubles fed to broiler chicks. J Sci Food Agric.

[bib26] Theander O., Aman P., Westerlund E., Andersson R., Pettersson D. (1995). Total dietary fiber determined as neutral sugar residues, uronic acid residues, and Klason lignin (the Uppsala method): collaborative study. J AOAC Int.

[bib27] Whiting I.M., Pirgozliev V., Rose S.P., Wilson J., Amerah A.M., Ivanova S.G., Staykova G.P., Oluwatosin O.O., Oso A.O. (2017). Nutrient availability of different batches of wheat distillers dried grains with solubles with and without exogenous enzymes for broiler chickens. Poult Sci.

[bib28] Widyaratne G.P., Zijlstra R.T. (2007). Nutritional value of wheat and corn distiller's dried grain with solubles: digestibility and digestible contents of energy, amino acids and phosphorus, nutrient excretion and growth performance of grower-finisher pigs. Can J Anim Sci.

